# Retraction: The Association of HMGB1 Gene with the Prognosis of HCC

**DOI:** 10.1371/journal.pone.0284595

**Published:** 2023-04-11

**Authors:** The PLOS ONE Editors

Following the publication of this article [1], concerns were raised regarding the results presented in Figs 3, 4, 6 and 7. Specifically,

- Lanes 1–4 of the Fig 3B HMGB1 panel appear similar to the Fig 4A HMGB1 panel (left) when rotated 180°
- The Fig 4A HMGB1 panel (right) appears similar to the Fig 4A Tubulin panel (right) when rotated 180°
- The Fig 3B and Fig 4A western blot results report Tubulin controls, but the article’s methodology section reports β-actin primers and antibodies instead.
- The following panels appear to partially overlap despite being used to represent different experimental conditions:
  ○ M6 (Fig 6), M6/mock (Fig 6), and M6/Mock (Fig 7)
  ○ M6/S2 (Fig 6) and M3 (Fig 7)

In addition to these concerns, it has come to the journal’s attention that several cell lines reported in this study have been reported to be misclassified or contaminated. Specifically,

- The HepG2 cell line reported in this study as a human hepatocellular carcinoma cell line has been misclassified, and should be reported as a hepatoblastoma cell line instead [2].
- The BEL-7404 cell line reported in this study as a human hepatocellular carcinoma cell line is a contaminated cell line, and has since the publication of this study been shown to be a HeLa derivative cell line instead [3].
- The L02 cell line reported in this study as a normal (non-cancerous) hepatocyte line is a contaminated cell line, and has since the publication of this study been shown to be a HeLa derivative cell line instead [4].

The first author commented that the image overlap was the result of errors during figure preparation and provided underlying data and updated figures. Furthermore, the first author stated that they omitted to include the use of tubulin in the methodology section, but this did not clarify why the study’s methodology reports on the use of β-actin instead, when no β-actin results have been presented in this study. In response to the contaminated cell line concerns, the first author states that the BEL-7404 and L02 cell lines were sent to a company for identification, but they did not provide further specification regarding the company or the identification procedure, and they did not provide evidence to confirm the cell lines used were not contaminated.

The first author provided underlying image data for the results presented in Figs 3, 4, 6, and 7, but the individual level data underlying the results in these figures were not provided for editorial review.

In light of the concerns affecting multiple figure panels that question the reliability of these image data, as well as the concerns pertaining to the contamination of cell lines including the control cell line that questions the reliability of the overall results, the *PLOS ONE* Editors retract this article.

JX did not agree with the retraction. YD, JH, QL, YL, WN, YZhang, YZhu, LC, and BC either did not respond directly or could not be reached.
